# Treatment discontinuation in HIV-1-infected individuals starting their first-line HAART after 2008: data from the ICONA Foundation Study Cohort

**DOI:** 10.7448/IAS.17.4.19825

**Published:** 2014-11-02

**Authors:** Antonio Di Biagio, Alessandro Cozzi-Lepri, Roberta Prinapori, Gioacchino Angarano, Andrea Gori, Tiziana Quirino, Andrea De Luca, Andrea Costantini, Cristina Mussini, Giuliano Rizzardini, Andrea Antinori, Antonella D'Arminio Monforte, on behalf of the ICONA, Foundation Study

**Affiliations:** 1 Department of Internal Medicine, San Martino Hospital, Genoa, Italy; 2 Division of Population Health, Department of Infection and Population Health, Royal Free Campus, UCL Medical School, London, UK; 3 Department of Internal Medicine, IRCCS AOU San Martino Hospital, Genoa, Italy; 4 Infectious Diseases, University of Bari, Bari, Italy; 5 Infectious Diseases, Hospital Monza, Monza, Italy; 6 Infectious Diseases, Hospital Busto Arsizio, Busto Arsizio, Italy; 7 Infectious Diseases, University of Siena, Siena, Italy; 8 Infectious Diseases, University of Ancona, Ancona, Italy; 9 Infectious Diseases, University of Modena, Modena, Italy; 10 Infectious Diseases, Sacco Hospital, Milano, Italy; 11 Infectious Diseases, INMI Lazzaro Spallaanzani, Roma, Italy; 12 Infectious Diseases, University of Milan, San Paolo Hospital, Milano, Italy; 13 Health Sciences, University of Milan, San Paolo Hospital, Milano, Italy

## Abstract

**Introduction:**

The aim of this study was to analyze the likelihood and the predictors of discontinuation of first-line regimen in the late HAART era.

**Methodology:**

An observational multi-center analysis of HIV-positive patients enrolled in ICONA. Patients eligible were those starting a first-line HAART after 1 January 2008. Discontinuation was defined as stop and/or switch of at least one drug of the regimen. All causes of discontinuation, as reported by the treating physician, were evaluated and cumulative risk of stopping was investigated according to age, gender, co-morbidity, years since starting HAART, immuno-virological status, third drug and backbone of the first regimen. Kaplan Meier (KM) analysis and Cox proportional hazards model were used for the outcome discontinuation of ≥1 drug regardless of the reason. For the KM estimates a competing risk approach was used to estimate the contribution of each of the reasons over time to the cumulative risk of stopping over time.

**Results:**

Data of 1759 patients who started first HAART and had at least one month of clinical follow-up were analyzed. The overall discontinuation risk was 33% over a median follow-up of 12 months. The likelihood of discontinuation by KM was 27% by one year (95% CI 25–29) and 41% by two years (95% CI 38–44). Main reason for stopping at least one drug in regimen was simplification (10%), followed by intolerance (7%), toxicity (5%), failure (2%) and other causes (8%). Estimates of the cumulative risk of discontinuation of ≥1 drug over time and according to reason are shown in Figure 1. In a multivariable Cox model independent predictors of discontinuation regardless of the reason were: longer time from HIV diagnosis to date of starting HAART (hazard ratio [HR] 0.96; 95% CI 0.93–1.00; p=0.039), regimens containing ZDV/3TC (HR 2.86; 95% CI 1.42–5.76; p=0.003 vs TDF/FTC) and an NNRTI-based regimen (HR 2.47; 95% CI 0.91–6.72; p=0.07 vs regimens not NNRTI-based).

**Conclusions:**

In a previously reported analysis of the ICONA data [1], the overall risk of discontinuation of first-line HAART was 36% with 21% due to intolerance/toxicity. In this updated analysis, the main reason for stopping is simplification (accounting for 32% of stops), reflecting the recent changes in recommendations aimed to minimize drug toxicity, enhancing adherence and quality of life.

**Figure 1:**
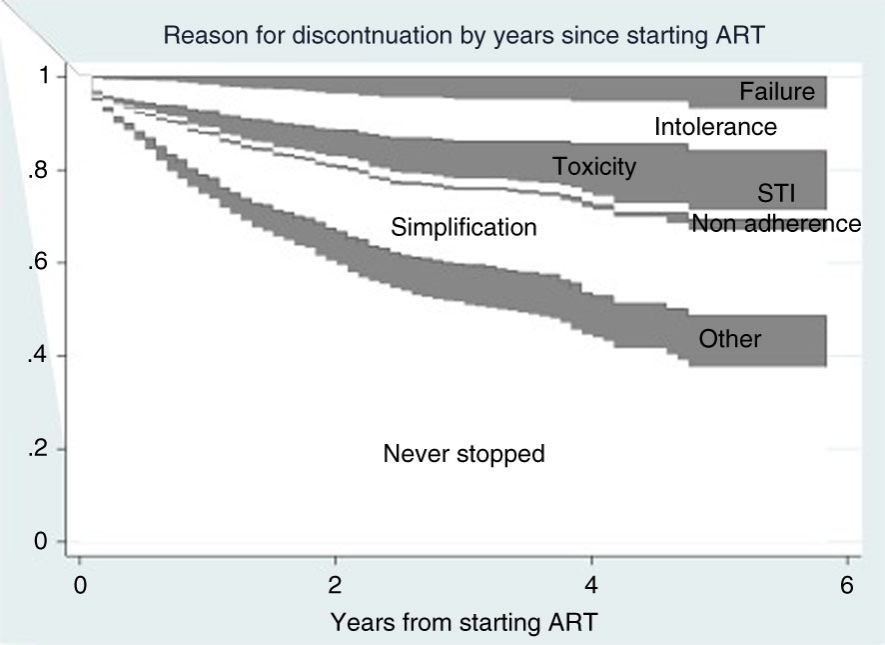
Reason of discontinuation.
